# Supplementary material for: Three-Dimensional Graphene Composite Containing Graphene-SiO2 Nanoballs and Its Potential Application in Stress Sensors
Source: Nanomaterials (Basel). 2019 Mar 15;9(3):438. doi: 10.3390/nano9030438 (PMC6474079; doi:10.3390/nano9030438)
Supplement: Supplementary file 1 [file nanomaterials-09-00438-s001.pdf]

Supporting Information

Three-dimensional graphene composite material doped with Grpahene-SiO_2 nanoballs and its potential application in stress sensors

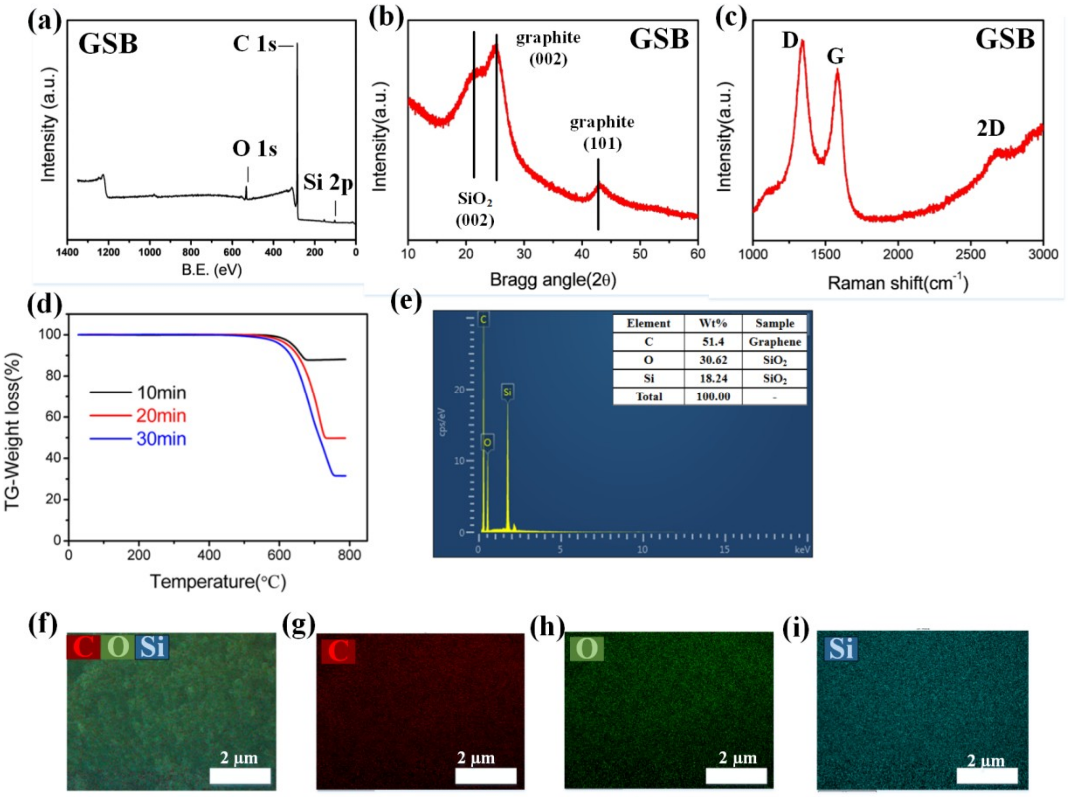

Figure S1. The characterization of GSB sample prepared with GO: SiO_2 ratio of 10:1 in 20 minutes (a) XPS spectrum of GSB (b) XRD spectrum of GSB (C) Raman spectrum of GSB. (D) TGA curve of GSB nanoparticles with different synthesis times. (e) Elemental content analysis of GSB. (f to i) EDS mapping of GSB in C, O, and Si elements, where C is assigned to graphene, and O and Si represent SiO_2.

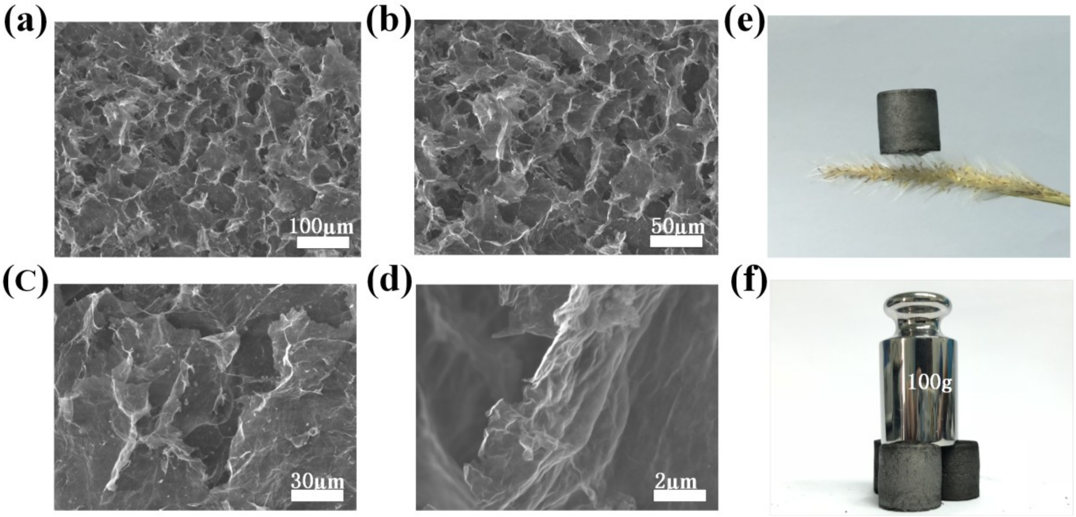

Figure S2. (a, b, c and d) SEM images of GSBF composites in different scales. (e) Photograph of GSBF sample which is light enough to stand on the tomentum. (f) Photograph of GSBF sample stressed by 100 g weight without visible strain.

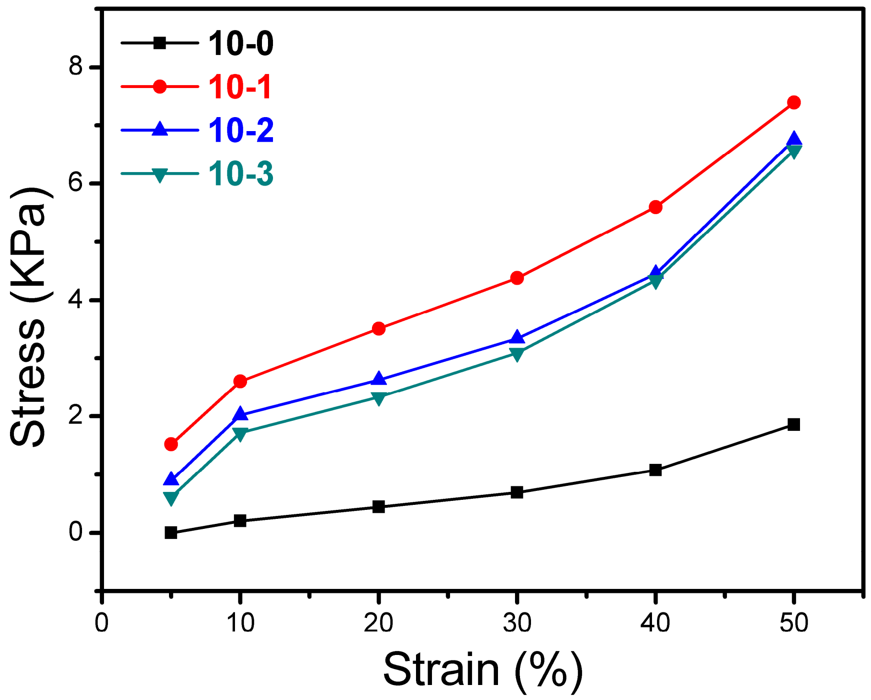

Figure S3. The stress-strain curves of GSBF with GO: GSB ratios of 10:0, 10:1, 10:2, and 10:3.

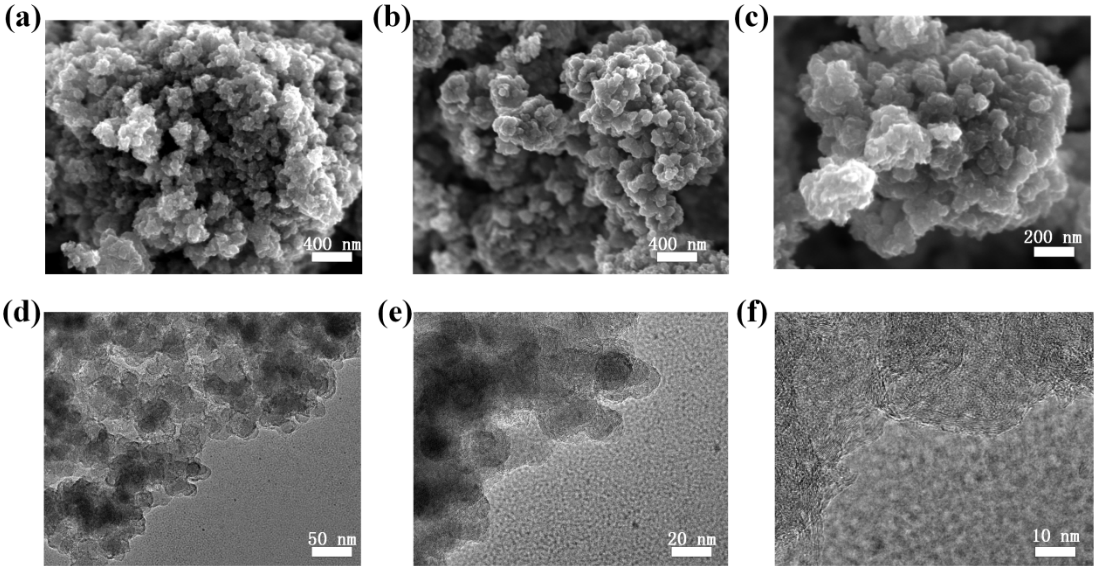

Figure S4. (a, b, c) SEM images of GSB sample. (d, e, f) TEM images of GSB sample.

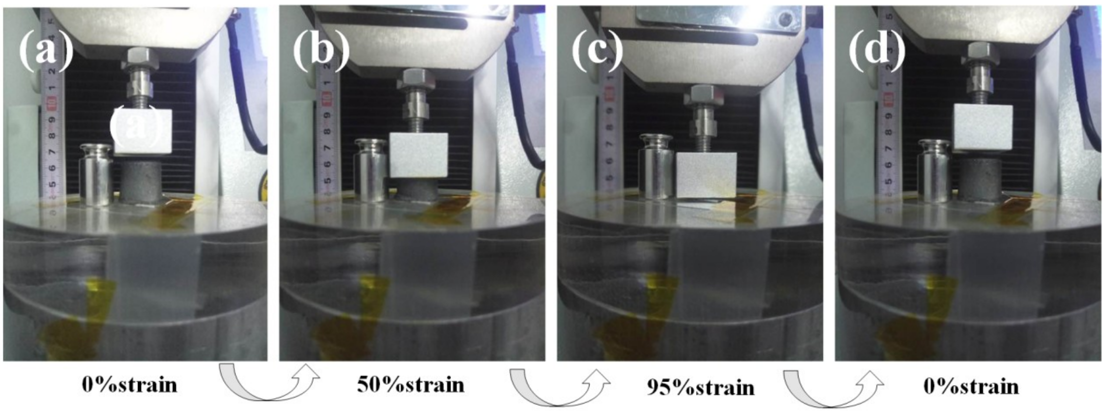

Figure S5. Photographs of GSBF material during the compression and recovery process with maximum strain of 95%.

Table S1. Properties of different graphene composite-based sensors

No.	Material	Stress range (kPa)	Sensitivity (kPa^{-1})	Ref.
1	Graphene foam	0-25	0.016	[1]
**2**	MWNT-rGO-PU foam	0-2.7	0.022	[2]
**3**	PAM hydrogel	0-3.2	0.05	[3]
**4**	PPy/CNT/PU	0-2	0.09	[4]
5	CNT/PDMS sponge	0-1	0.1	[5]
**6**	CB-PU	0-2.3	0.068	[6]
7	VACNT/PDMS	0-2	0.05	[7]
8	GF/CNT	0-2.5	0.19	[8]
9	rGO/PI	0-1.5	0.18	[9]
10	GO/PS/SDS	0-1.5	0.22	[10]
**11**	**This work**	0-10	0.14	-
		15-30	0.03	

Reference

1. Yang, J.; Li, X.; Lu, X.; Bao, W.; Chen, R. Three-Dimensional Interfacial Stress Sensor Based on Graphene Foam. *IEEE Sensors Journal* **2018**, *18*, 7956-7963, doi:10.1109/jsen.2018.2855691.
2. Tewari, A.; Gandla, S.; Bohm, S.; McNeill, C.R.; Gupta, D. Highly Exfoliated MWNT-rGO Ink-Wrapped Polyurethane Foam for Piezoresistive Pressure Sensor Applications. *ACS Appl Mater Interfaces* **2018**, *10*, 5185-5195, doi:10.1021/acsami.7b15252.
3. Ge, G.; Zhang, Y.; Shao, J.; Wang, W.; Si, W.; Huang, W.; Dong, X. Stretchable, Transparent, and Self-Patterned Hydrogel-Based Pressure Sensor for Human Motions Detection. *Advanced Functional Materials* **2018**, *28*, doi:10.1002/adfm.201802576.
4. da Silva, F.A.G.; de Araújo, C.M.S.; Alcaraz-Espinoza, J.J.; de Oliveira, H.P. Toward flexible and antibacterial piezoresistive porous devices for wound dressing and motion detectors. *Journal of Polymer Science Part B: Polymer Physics* **2018**, *56*, 1063-1072, doi:10.1002/polb.24626.
5. Yu, G.; Hu, J.; Tan, J.; Gao, Y.; Lu, Y.; Xuan, F. A wearable pressure sensor based on ultra-violet/ozone microstructured carbon nanotube/polydimethylsiloxane arrays for electronic skins. *Nanotechnology* **2018**, *29*, 115502, doi:10.1088/1361-6528/aaa855.
6. Wu, X.; Han, Y.; Zhang, X.; Zhou, Z.; Lu, C. Large-Area Compliant, Low-Cost, and Versatile Pressure-Sensing Platform Based on Microcrack-Designed Carbon Black@Polyurethane Sponge for Human-Machine Interfacing. *Advanced Functional Materials* **2016**, *26*, 6246-6256, doi:10.1002/adfm.201601995.
7. Kang-Hyun Kim; Soon Kyu Hong; Nam-Su Jang; Sung-Hun Ha; Hyung-Woo Lee; Kim., J.-M. Wearable Resistive Pressure Sensor Based on Highly Flexible Carbon Composite Conductors with Irregular Surface Morphology. *ACS Appl Mater Interfaces* **2017**, *9*, 17500-17508.
8. Kuang, J.; Dai, Z.; Liu, L.; Yang, Z.; Jin, M.; Zhang, Z. Synergistic effects from graphene and carbon nanotubes endow ordered hierarchical structure foams with a combination of compressibility, super-elasticity and stability and potential application as pressure sensors. *Nanoscale* **2015**, *7*, 9252-9260, doi:10.1039/c5nr00841g.
9. Yuyang Qin; Qingyu Peng; Yujie Ding; Zaishan Lin; Chunhui Wang; Ying Li, F.X.; Jianjun Li; Ye Yuan; Xiaodong He; Li., Y. Lightweight, Superelastic, and Mechanically Flexible Graphene/Polyimide Nanocomposite Foam for Strain Sensor Application. *ACS Nano* **2015**, *9*, 8933-8941.
10. Zhang, P.; Lv, L.; Cheng, Z.; Liang, Y.; Zhou, Q.; Zhao, Y.; Qu, L. Superelastic, Macroporous Polystyrene-Mediated Graphene Aerogels for Active Pressure Sensing. *Chem Asian J* **2016**, *11*, 1071-1075, doi:10.1002/asia.201600038.
